# Managing Institutional Diversity in Integrated Care: A Systematic Literature Review of Institutional Logics and Management Strategies

**DOI:** 10.5334/ijic.8587

**Published:** 2025-03-27

**Authors:** Marco Roth

**Affiliations:** 1 Public Financial Management, Tampere University’s Faculty of Management and Business, FI; 2 Wellbeing Services County of Pirkanmaa, FI

**Keywords:** integrated care, management strategies, institutional logics, hybrid governance, institutional hybridity

## Abstract

**Introduction:**

This review examines the institutional diversity in integrated care due to various institutional logics and explores the essential management strategies for navigating this diversity effectively.

**Methods:**

This systematic literature review involved a comprehensive search across Scopus, Medline and Google Scholar. This process identified 1747 articles, with rigorous screening narrowing them down to eight key articles.

**Results:**

The study unveils five primary institutional logics in integrated care, focusing on their unique characteristics and how they interrelate, and highlights crucial management strategies for maintaining a balance among these diverse logics.

**Discussion:**

Applying the institutional logic perspective, this study uncovers the complex interplay of diverse logics shaping integrated care—a previously underexamined facet. Examining the foundational beliefs underlying organizational behaviour offers deeper insights into institutional complexities often overlooked in previous literature. This highlights the necessity for effective management strategies to reconcile conflicting logics, promoting more sustainable integrated care solutions.

**Conclusion:**

The review comprehensively explores institutional logics in integrated care, emphasizing the importance of holistic management strategies. It highlights the imperative for policymakers and healthcare administrators to balance operational efficiency, patient-focused care, and professional independence—essential elements in crafting successful integrated care frameworks.

## Introduction

The concept of integrated care has emerged as a pivotal response to the evolving complexities in the realms of social and healthcare. In an era marked by rapid societal, economic and healthcare transformations, integrated care [1] stands at the forefront of a paradigm shift. This comprehensive approach seeks to unify various facets of social and healthcare delivery, reorienting them towards a more collaborative, efficient and patient-centric model. It addresses the growing need for cohesiveness in an increasingly fragmented social care and healthcare landscape, a demand fuelled by demographic changes, technological advancements and shifting patient expectations. The evolution of integrated care is rooted in both historical developments and theoretical underpinnings, with cross-sector collaboration, patient-centredness and efficiency emphasised as central tenets [2].

However, integrating care systems is challenged by the need to navigate diverse institutional logics, which provide a theoretical framework offering deeper insights into the underlying principles shaping how individuals, groups, and organizations operate within the social and healthcare sector. Institutional logics are defined as socially constructed patterns of practices, assumptions, values, beliefs, and rules through which individuals and organizations provide meaning to their social reality and material life [3,4]. Understanding individual and organizational behaviour necessitates locating it within a social and institutional context, as this context both regularises behaviour and provides opportunities for agency and change [4]. Introducing institutional logics into the discussion on integrated care enhances our understanding of the complexities and inherent contradictions influencing its implementation.

In this context, institutional logics encompass the varied and sometimes conflicting principles that guide social and healthcare organizations, including professional norms, regulatory standards, and patient care philosophies. Integration is not merely practical but involves reconciling these diverse logics to create a cohesive system. The relevance of institutional logics to integrated care lies in their profound influence on organizational structures, practices, and stakeholder interactions within the social and healthcare system [5]. Despite the recognized importance of integrated care, a gap persists in academic research concerning the interplay of institutional logics [5,6] and the development of effective management strategies [7] to navigate their diversity. Establishing a theoretical framework based on institutional logics allows for deeper exploration of how these differing logics can both hinder and facilitate integrated care efforts. Incorporating institutional logics uncovers deeper institutional forces and contradictions that practical strategies alone may not reveal, offering novel insights into the challenges of integrated care.

Therefore, this literature review is driven by two critical research questions (RQs):

RQ1. What are the key institutional logics present in integrated care, and what are the nature of their synergies and conflicts?

RQ2. What are the management strategies for navigating the diversity of institutional logics in integrated care?

By addressing these questions, this review aims to dissect the roles and impacts of institutional logics and management strategies in integrated care. Building upon a theoretical framework grounded in institutional logics, the study articulates how these logics are integral to understanding and implementing integrated care. This approach contributes to the integrated care discourse by providing a theoretical framework that has received little attention in previous literature, thereby enhancing our understanding of the complexities involved. The goal is to provide a comprehensive analysis of the interplay between institutional logics and management strategies, offering fresh perspectives and practical insights for managing institutional diversity. Ultimately, this review endeavours to foster the development of more adaptable, efficient and patient-centric healthcare systems, enriched by a thorough understanding of the intricate institutional landscape that shapes contemporary social and healthcare delivery.

## Methodology

### Systematic Literature Review

This study utilised a systematic literature review (SLR), a fundamental method in academic research that is known for providing a structured overview of existing knowledge. This methodology is adept at exploring complex phenomena, such as the interplay of institutional logics in integrated care and their management strategies. As outlined [8,9], the SLR process involves defining clear RQs, setting precise criteria for study selection and comprehensively searching databases. This structured and transparent approach, further reinforced by the Preferred Reporting Items for Systematic Reviews and Meta-Analyses (PRISMA) guidelines [10,11], ensures a researcher’s thorough exploration and synthesis of the literature, thereby providing reliable insights and highlighting knowledge gaps in the realm of integrated care.

### Search Strategy

The literature search leveraged the broad multidisciplinary scope of Scopus, Medline and Google Scholar [12]. The search terms included “integrated care” OR “integrated health” OR “coordinated care” OR “comprehensive care” OR “seamless care” OR “transmural care” OR “disease management” OR “care management” OR “managed care” AND “institutional logics” OR “institutional logic”. Initially, the search yielded 1,747 articles (November 2023), refined by removing duplicates and non-journal materials. Screening of titles, abstracts, and keywords to ensure both study’s key concepts—integrated care or its synonyms and institutional logics—were mentioned, narrowed the selection to 54 articles. These were reviewed through a double-blind process with a peer colleague, ensuring alignment with the study’s RQs, particularly the interplay of multiple institutional logics within integrated care. Articles focused on a single institutional logic or possessing predominantly identical authorship and analogous research frameworks regarding institutional logics were excluded. Inclusion criteria required publication in peer-reviewed scientific journals (with a 5-year Impact Factor mean of 2.713 for selected articles’ journals and articles’ mean citation count >21). This process resulted in the selection of eight articles for qualitative analysis, chosen for their relevance to the research questions and insights into institutional logics in integrated care settings. A summary of the screening and selection process is provided in Table 1. The peer reviewer concurred with all but one selection, leading to a consensus through discussion.

**Table 1:** Study Selection Flowchart.


STAGE	DETAILS	TOTAL ARTICLES

**Identification**	PubMed/Medline = 7; Scopus = 768; Google Scholar = 972	1747

**Screening**	Duplicates and non-journal materials removed (n = 965)	782

**Eligibility**	Titles, abstracts, and keywords were screened, excluding 728 articles that did not mention both key concepts: integrated care or its synonyms and institutional logics.	54

**Inclusion**	Full-text articles assessed for eligibility, excluding (n = 46) non-peer-reviewed articles, topics that did not primarily focus on both concepts, covered a single institutional logic or were broadly similar in research design, conducted by the same authors. Final articles included for qualitative analysis (n = 8)	8


### Data Extraction and Analysis

The qualitative content analysis method [13] was used to interpret and categorise the textual data from the selected articles through iterative coding to identify key concepts, themes, and patterns relevant to institutional logics and management strategies in integrated care. The analysis drew on approaches used [14,15,16] to understand the dynamics of institutional logics and the strategic responses in managing institutional diversity within integrated care settings.

Data extraction focused on specific information from each article, aligned with RQs. This entailed a thorough examination of each study’s methodology, findings and conclusions. Key aspects such as study design, contextual settings, the nature of institutional logics and the employed management strategies were extracted. This enabled the distillation of insights directly pertinent to the research inquiries. For instance, exploring the interactions among various institutional logics in integrated care settings (RQ1) revealed how these logics manifested in practice and influenced care delivery outcomes. Similarly, addressing management strategies (RQ2) involved obtaining detailed descriptions, implementation contexts, and effectiveness. This approach was pivotal in constructing a cohesive narrative from the disparate studies, enriching the understanding of the complexities in managing institutional diversity.

The selected articles underwent comparative analysis [17] to identify commonalities, differences, trends and anomalies in the manifestation and management of institutional logics in integrated care. This analytical step was vital in developing a comprehensive understanding of the field and pinpointing gaps in existing literature.

### Data Organisation

The data organisation began with the initial coding [18], involving an exhaustive examination of the text to identify codes representing concepts, themes or categories pertinent to institutional logics and management strategies in integrated care. Both a priori codes from existing literature [19] and in vivo codes from the data emerged. These initial codes were aggregated into broader categories, following the thematic categorisation framework [20], highlighting patterns that resonate with the RQs and objectives.

Matrices and summaries [21] were used to organise the data, facilitating the comparison of themes and patterns across different studies and ensuring a structured synthesis of the findings.

## Results

### Institutional Logics and Their Interactions in Integrated Care

The systematic literature review identified key themes and patterns in how institutional logics—managerial, professional, patient-centric, market, and state—interact within integrated care settings. These logics and their key interactions are summarized in Table 2. Notably, several tensions and contradictions emerged from these interactions. A prominent tension exists between managerial logic’s emphasis on efficiency and cost-effectiveness and professional logic’s prioritization of clinical autonomy and quality of care. Managerial imperatives for standardization and resource optimization often clash with professionals’ desire for discretion in clinical decision-making [22,23,24,25,26,27].

**Table 2:** Institutional Logics and Their Interactions in Integrated Care.


INSTITUTIONAL LOGIC	DESCRIPTION	KEY SYNERGIES AND CONFLICTS	REFERENCES

**Managerial**	Focuses on efficiency, cost-effectiveness, resource management and evidence-based practices	Conflicts: Contradicts patient-centric logic when efficiency compromises individualized care; clashes with professional logic over standardization vs. clinical autonomy Synergies: Aligns with market logic in promoting innovation and efficiency	Kokko & Laihonen [22]; Mæhle et al. [23]; Mansfield et al. [24]; Allen [25]; Dent & Tutt [26]; Novotná [27]

**Professional**	Emphasises medical expertise and clinical autonomy, integrating clinical research for healthcare quality improvement	Conflicts: Tensions with managerial logic over efficiency measures; challenges with state logic when regulations limit autonomy Synergies: Aligns with patient-centric logic in prioritizing patient needs	Kokko & Laihonen [22]; Oksavik et al. [6]; Mæhle et al. [23]; Mansfield et al. [24]; Goodrick & Reay [28]; Dent & Tutt [26]; Novotná [27]

**Patient-centric**	Prioritises individual patient needs and personalisation of care	Conflicts: Overshadowed by managerial efficiency demands and professional standard practices Synergies: Supports professional logic when personalized care is valued	Kokko & Laihonen [22]; Mæhle et al. [23]

**State**	Aligns with government policies, public health goals and regulatory compliance	Conflicts: Regulatory requirements may limit professional autonomy; policy objectives may conflict with market-driven approaches Synergies: Supports managerial logic through standardization mandates	Goodrick & Reay [28]; Oksavik et al. [6]

**Market**	Represents a business-oriented approach, focusing on competition, efficiency and consumer choice	Conflicts: May conflict with state regulations aimed at equity; challenges patient-centric care when profit motives predominate Synergies: Aligns with managerial logic in efficiency and innovation	Goodrick & Reay [28]; Oksavik et al. [6]; Novotná [27]


While patient-centric logic theoretically aligns with both managerial and professional logics, it frequently conflicts with efficiency demands and standardized practices, challenging the delivery of personalized care. The push for efficiency can lead to reduced time for patient interaction, undermining the patient-centered approach that values individual needs and preferences [22,23]. The impact of market logic introduces additional complexity. Market logic encourages innovation and efficiency but may conflict with state logic’s focus on equitable public health outcomes, introducing competitive pressures that affect resource allocation and access to care. This can create disparities and tensions between profitability and the provision of universal care services [27,28,6]. State logic, encompassing government policies and regulations, plays a dual role by both facilitating and constraining integrated care implementation. While it supports standardization and alignment with public health goals, it may also limit professional autonomy and flexibility, creating friction with professional and managerial logics [28,6].

These themes highlight inherent contradictions within integrated care systems, as depicted in Table 2. The pursuit of standardized processes under managerial logic may overlook individual patient needs and professional expertise valued by patient-centric and professional logics, leading to a conflict between standardization and personalization. Similarly, efficiency drives may compromise the quality of care, exemplifying the tension between efficiency and quality that creates friction between managerial objectives and professional standards.

These findings contribute new insights by elucidating the complex interplay of institutional logics within integrated care—a dimension less explored in existing literature. By highlighting these specific tensions and contradictions, the study extends the understanding of the institutional dynamics that influence the success of integrated care initiatives.

Understanding these interactions is crucial for developing management strategies that balance competing demands. Recognizing the necessity to navigate tensions such as efficiency versus personalization and regulation versus autonomy allows administrators to design interventions that address these institutional dynamics. This underscores the importance of strategic approaches attuned to the multifaceted institutional landscape of integrated care, ultimately contributing to more effective and sustainable healthcare systems.

### Management Strategies for Coping with Institutional Diversity in Integrated Care

The systematic literature review revealed specific themes and patterns in management strategies employed to navigate institutional diversity in integrated care. These strategies are crucial for reconciling divergent institutional logics—managerial, professional, patient-centric, market, and state—to enhance the efficacy and coherence of social and healthcare delivery systems. Table 3 summarizes these strategies, their benefits, challenges, and application examples.

**Table 3:** Management Strategies for Institutional Diversity in Integrated Care.


MANAGEMENT STRATEGIES	DESCRIPTION	BENEFITS	CHALLENGES	APPLICATION EXAMPLES	REFERENCES

**Hybrid organisational structures and collaborative governance**	Incorporates and synthesises diverse logics to foster new hybrid organisational structures and enhance collaborative governance in social and healthcare	Fosters trust and shared understanding Reconciles different institutional perspectives	Complexity in integrating diverse logics Optimizing coordination and communication in complex hybrid structures	Healthcare alliance model engaging diverse institutional providers Multidisciplinary healthcare teams implementing shared governance	Kokko & Laihonen [22]; Mæhle et al. [23]; Allen [25]

**Performance management and accountability**	Enforces a balanced approach to performance management that acknowledges and integrates various institutional logics for improved accountability	Promotes fact-based decision-making Aligns with diverse stakeholder requirements	Balancing conflicting stakeholder interests Maintaining transparency and accountability	Use of performance metrics in healthcare institutions Data-driven clinical decision-making	Kokko & Laihonen [2]); Dent & Tutt [26]; Allen [25]

**Patient-centric strategies and care coordination**	Prioritises patient-centric logic in integrated care strategies to ensure that care delivery is coordinated and aligns with patients’ multifaceted needs	Aligns care with patient preferences Fosters empathy and inclusivity	Integrating patient perspectives with institutional goals Complexity in coordinating care	Personalised patient care plans in a hospital setting Patient advocacy in treatment decisions	Oksavik et al. [26]; Mæhle et al. [23]; Allen [25]

**Innovative practices and frontline engagement**	Champions innovation by aligning frontline practices with the complex interplay of managerial, professional and patient-centred logics	Encourages responsive and adaptable care Utilises unique frontline insights	Challenges in fostering a culture of innovation Managing changes at the frontline	Frontline staff-led initiatives in a clinical setting Staff-driven process optimisation initiatives	Mansfield et al. [24]; Goodrick & Reay [28]; Dent & Tutt [26]

**Systemic and strategic transformation**	Undertakes systemic transformation by strategically aligning managerial, professional and market logics with the dynamic landscape of patient care	Ensures long-term adaptability Responsive to evolving healthcare needs	Complexity of systemic transformation Aligning diverse frameworks with care demands	Overhaul of policy and financial frameworks in a healthcare organisation Regulatory framework revision	Novotná [27]; Mansfield et al. [24]; Mæhle et al. [23]


One prominent theme is the development of hybrid organizational structures and collaborative governance to integrate diverse institutional logics. This approach involves creating novel organizational models that transcend traditional boundaries, fostering trust and mutual understanding among stakeholders. For instance, Kokko and Laihonen [22] highlighted how multi-organizational alliances in healthcare blend public, private, and third-sector entities, forming hybrid models that reconcile managerial efficiency with professional autonomy and patient-centric values. This strategy addresses the contradiction between the need for standardized management practices and the preservation of professional expertise, demonstrating that collaborative structures can facilitate the harmonization of conflicting logics.

Another theme is the implementation of performance management and accountability systems that acknowledge and integrate various institutional logics. By utilizing integrated databases and performance metrics, organizations promote transparent, fact-based decision-making that aligns with diverse stakeholder requirements. Allen [25] illustrated how aligning nursing and managerial interests through performance management enhances care coordination and service quality, integrating managerial, professional, and patient-centric logics. This strategy, however, must navigate the contradiction of balancing conflicting stakeholder interests while maintaining transparency and accountability.

The emphasis on patient-centric strategies and care coordination emerged as a critical pattern, prioritizing patient empowerment and individualized care plans. Oksavik et al. [6] demonstrated how personalized care plans effectively amalgamate managerial, professional, and patient-centric logics, addressing patients’ multifaceted needs. This approach confronts the challenge of integrating patient perspectives with institutional goals, highlighting the tension between organizational efficiency and personalized care. By fostering empathy and inclusivity, this strategy contributes to a more holistic and patient-responsive integrated care system.

Innovative practices and frontline engagement represent a strategy that empowers frontline staff to align practices with the complex interplay of institutional logics. Studies by Mansfield et al. [24] and Goodrick and Reay [28] showcased how frontline initiatives, such as integrating mental and physical health services, disrupt traditional workflows and enhance patient-provider relationships. This strategy addresses the contradiction between established institutional practices and the need for innovation, demonstrating that frontline engagement can facilitate adaptability and responsiveness in care delivery.

Lastly, the theme of systemic and strategic transformation involves overhauling policy and financial frameworks to align managerial, professional, and market logics with evolving patient care needs. Novotná [27] exemplified this approach through the amalgamation of mental health and addiction treatment organizations in Ontario, aiming for cost-effectiveness and coordinated services. This strategy underscores the necessity of long-term adaptability but faces the challenge of aligning diverse frameworks and managing the complexity of systemic transformation.

These findings contribute new insights by detailing how specific management strategies address the interplay of institutional logics within integrated care—a dimension that extends existing knowledge in the field. By identifying themes such as the balance between standardization and personalization, efficiency and quality, and innovation and tradition, the study highlights the critical role of strategic management in navigating institutional diversity. Understanding these patterns and contradictions informs the development of effective management interventions that can harmonize competing logics, ultimately enhancing the sustainability and effectiveness of integrated healthcare systems.

The implications of these strategies are significant for practitioners and policymakers. By adopting approaches that integrate diverse institutional logics, organizations can improve collaboration, accountability, patient-centricity, innovation, and adaptability in integrated care settings. Recognizing and strategically managing the inherent tensions between different logics is essential for the successful implementation and optimization of integrated care initiatives.

## Discussion

This SLR illuminates the multifaceted nature of institutional logics in the realm of integrated care and identifies a spectrum of management strategies to navigate this diversity. By applying the institutional logic framework, this study provides deeper theoretical insights into the complexities of integrated care that have been given too little attention in previous literature. The findings reveal that integrated care is not a monolithic concept but a confluence of various institutional logics (RQ1). Each logic brings its unique perspective and imperative, shaping the integrated care landscape in distinct ways. The revelation of integrated care as a confluence of multiple logics signifies that practitioners and policymakers must recognize and navigate these overlapping logics to implement effective integrated care models. This complexity challenges the assumption of integrated care as a straightforward amalgamation of services, highlighting instead the intricate balancing act required between conflicting logics.

The interplay of these logics underscores the complexity inherent in integrated care. For instance, managerial logic, focusing on operational efficiency and cost-effectiveness, can occasionally conflict with patient-centric logic that values individualised care and patient preferences. In integrated care, the interactions among these logics are more pronounced than those reported in the general social and healthcare literature, where the interplay of managerial and professional logics prevails, and patient centrality is typically manifested through the lens of professional logic (see, e.g., [29,30]). Similarly, the interactions between state and professional logics are evident in integrated care, a phenomenon that is less commonly observed in the general social and healthcare literature (see [31,6]). This amplification of institutional complexity in integrated care necessitates a more nuanced understanding and tailored management strategies to effectively reconcile conflicting imperatives.

This juxtaposition in integrated care settings highlights a distinct dynamic, where the regulatory and policy-driven imperatives of state logic intersect with and sometimes challenge the clinical discretion of professional logic. These findings support the institutional logic framework’s assertion that organizations operate within multiple, sometimes competing logics, emphasizing the need for strategies that accommodate such pluralism. By demonstrating how integrated care settings intensify the interplay of various logics, the study extends the theoretical application of institutional logics within social and healthcare contexts.

This review advances the academic discourse on integrated care by situating the concept within the framework of institutional logics—an approach less explored in existing literature. By doing so, it fills gaps in understanding, offering explanations for challenges not fully examined in prior studies. The identified institutional logics and their interactions align with literature emphasizing the heterogeneity and complexity of social and healthcare systems (e.g., [32,33]). By providing a more comprehensive understanding of how these logics coexist and interact within integrated care, this review enriches the existing body of knowledge. Moreover, the findings corroborate theories advocating for pluralistic approaches in organizational management, highlighting the necessity of embracing institutional diversity to enhance integrated care practices.

The management strategies (RQ2) identified in this review, while diverse, collectively underscore a transformative approach to integrated care. Understanding institutional logics aids practitioners and policymakers in managing institutional diversity and contradictions by informing these strategies. These strategies, spanning hybrid organisational structures and collaborative governance (cf. [34,35]), performance management and accountability (cf. [36,37]), patient-centric strategies (cf. [38]), innovative practices and frontline engagement (cf. [39]), and systemic and strategic transformation (cf. [40]), highlight a unified theme of adaptability and holistic integration. They emphasise the importance of multidisciplinary collaboration and patient-centric approaches, aligning with the contemporary push towards more responsive and personalised integrated care. Critically, these management strategies illustrate practical means by which organizations can navigate the pluralistic institutional environment of integrated care. For practitioners, this underscores the necessity of developing competencies in inter-professional collaboration, flexibility, and the ability to operate within and across multiple institutional logics. Policymakers are impelled to design policies that recognize and support the coexistence of diverse logics, facilitating structures that promote collaboration without undermining professional autonomy. These findings emphasize the importance of leadership in mediating conflicting logics and fostering an organizational culture that values diversity and adaptability.

These strategies also advocate continuous innovation and improvement, underlining the dynamic nature of integrated care delivery. This comprehensive approach is crucial for effectively navigating the complexities and evolving demands of integrated care systems, ensuring that they are equipped to meet the diverse needs of patients and social and healthcare professionals alike.

While this review offers valuable insights, it is important to acknowledge its limitations. First, it may be subject to publication bias because studies reporting positive outcomes or novel findings are more likely to be published. Second, the review predominantly relies on literature written in English, potentially overlooking relevant studies published in other languages. Third, the dynamic and rapidly evolving healthcare field may mean that newer developments and emerging logics are not fully captured in this review.

To advance integrated care, future research should systematically examine how different institutional logics interact in specific settings, revealing strategies for managing hybrid logics and effectively handling institutional complexity. Analyzing how these interactions affect patient care outcomes is essential. Future studies could also explore the role of leadership and organizational culture in mediating these interactions, providing insights into effective management practices in integrated care settings. Rigorous longitudinal studies are needed to assess the enduring impacts of management strategies and their resilience amid healthcare’s rapid evolution. Such inquiry ensures integrated care systems are not only effective in the short term but also sustainable and adaptable for the future, leading to more cohesive and patient-responsive social and healthcare ecosystems.

## Conclusion

This SLR provides a comprehensive analysis of managing institutional diversity in integrated care by examining the interplay between various institutional logics and management strategies. The findings highlight the necessity of adept strategies that reconcile these logics—from managerial efficiency to patient-centered care—to foster a responsive and sustainable integrated care ecosystem. Emphasizing adaptability and comprehensive integration, the review advocates balanced approaches that ensure operational efficiency, uphold patient-centered care, and maintain professional autonomy. These insights are crucial for policymakers and healthcare administrators as they develop effective integrated care models to meet the complex demands of today’s social and healthcare landscape. By enhancing the understanding of integrated care’s multifaceted nature, the review underscores the importance of adaptive management and nuanced strategies that consider the unique contributions of different institutional logics to improve patient care and organizational performance.
